# Generic phylogeny, historical biogeography and character evolution of the cosmopolitan aquatic plant family Hydrocharitaceae

**DOI:** 10.1186/1471-2148-12-30

**Published:** 2012-03-10

**Authors:** Ling-Yun Chen, Jin-Ming Chen, Robert Wahiti Gituru, Qing-Feng Wang

**Affiliations:** 1 CAS Key Laboratory of Aquatic Botany and Watershed Ecology, Chinese Academy of Sciences, Wuhan 430074, Hubei, P. R. China; 2 Wuhan Botanical Garden, Chinese Academy of Sciences, Wuhan 430074, Hubei, P. R. China; 3 Graduate University of Chinese Academy of Sciences, 100049 Beijing, China; 4 Botany Department, Jomo Kenyatta University of Agriculture and Technology, P. O. Box 62000-00200, Nairobi, Kenya

**Keywords:** Hydrocharitaceae, Phylogeny, Historical biogeography, Dispersal, Vicariance, Morphological character evolution

## Abstract

**Background:**

Hydrocharitaceae is a fully aquatic monocot family, consists of 18 genera with approximately 120 species. The family includes both fresh and marine aquatics and exhibits great diversity in form and habit including annual and perennial life histories; submersed, partially submersed and floating leaf habits and linear to orbicular leaf shapes. The family has a cosmopolitan distribution and is well represented in the Tertiary fossil record in Europe. At present, the historical biogeography of the family is not well understood and the generic relationships remain controversial. In this study we investigated the phylogeny and biogeography of Hydrocharitaceae by integrating fossils and DNA sequences from eight genes. We also conducted ancestral state reconstruction for three morphological characters.

**Results:**

Phylogenetic analyses produced a phylogeny with most branches strongly supported by bootstrap values greater than 95 and Bayesian posterior probability values of 1.0. *Stratiotes *is the first diverging lineage with the remaining genera in two clades, one clade consists of *Lagarosiphon, Ottelia, Blyxa, Apalanthe, Elodea *and *Egeria*; and the other consists of *Hydrocharis*-*Limnobium, Thalassia, Enhalus, Halophila, Najas, Hydrilla, Vallisneria, Nechamandra *and *Maidenia*. Biogeographic analyses (DIVA, Mesquite) and divergence time estimates (BEAST) resolved the most recent common ancestor of Hydrocharitaceae as being in Asia during the Late Cretaceous and Palaeocene (54.7-72.6 Ma). Dispersals (including long-distance dispersal and migrations through Tethys seaway and land bridges) probably played major roles in the intercontinental distribution of this family. Ancestral state reconstruction suggested that in Hydrocharitaceae evolution of dioecy is bidirectional, viz., from dioecy to hermaphroditism, and from hermaphroditism to dioecy, and that the aerial-submerged leaf habit and short-linear leaf shape are the ancestral states.

**Conclusions:**

Our study has shed light on the previously controversial generic phylogeny of Hydrocharitaceae. The study has resolved the historical biogeography of this family and supported dispersal as the most likely explanation for the intercontinental distribution. We have also provided valuable information for understanding the evolution of breeding system and leaf phenotype in aquatic monocots.

## Background

Hydrocharitaceae is a fully aquatic monocot family, consists of 18 genera and approximately 120 species [1,2] with a cosmopolitan distribution. The group is an important component of aquatic ecosystems and occurs in both freshwater and marine habitats. It also exhibits great diversity in form and habit including annual and perennial life histories; submersed, partially submersed and floating leaf habits, and linear to orbicular leaf shapes [1]. It includes a number of valuable aquarium plants and some species (e.g., *Hydrilla verticillata *and *Elodea canadensis*) provide fish breeding sites and fodder for the poultry raising industry [3]. Owing to habitat destruction and unprecedented increase in commercial shipping activities, several species (e.g., *Ottelia alismoides *and *Blyxa japonica*) are threatened, while some other species (e.g., *Hydrilla verticillata*) have become invasive weeds of great concern [4]. Similar to many aquatic plants, the group displays considerable convergent adaptations and morphological reduction, which make the study of phylogenetics and taxonomy of the group difficult [1,5-7]. There exists little consensus in classification systems of the group based on morphological characters [1,8].

Although molecular phylogenetic studies of Hydrocharitaceae have created consensus on the relationships between certain genera, disagreements on the relationships of other genera still exist. The seagrass subclade which includes *Halophila, Enhalus *and *Thalassia *was resolved as sister to the subclade comprising *Najas, Hydrilla *and *Vallisneria *by analyses using *rbc*L+ *mat*K [8] and *rbc*L+ *cob *+ *atp*1 [9]. However, the seagrasses was resolved as sister to the subclade comprising *Nechamandra, Vallisneria *and *Maidenia *by analyses using morphological + *rbc*L+ *mat*K+ *trn*K intron + ITS [1]. Similarly, *Hydrilla*, which was reported to be closely related to *Vallisneria *by analyses using *rbc*L, *mat*K [2,8], *rbc*L, *cob *and *atp*1 [9], was reported to be closely related to *Najas *by analyses using morphological character + *rbc*L+ *mat*K+ *trn*K intron + ITS [1]. Furthermore, *Stratiotes *has been resolved as sister to the rest of this family by analyses using *rbc*L [9] and mitochondrial genes (*nad*5, *cob, ccm*B, *mtt*2, *atp*1) [9,10]. The position is not in agreement with the results of two other studies using chloroplast and nuclear sequences [1,2]. In addition, a single species was selected for each genus in previous studies. The phylogeny of this family could be improved by a better sampling of taxon and DNA sequence, and perhaps a careful outgroup selection [1].

The divergence time of Hydrocharitaceae is still a subject of debate, and two competing ages (one much more recent than the other) have been proposed. Janssen and Bremer (2004) [11] placed the crown node age of this family in the Late Cretaceous (75 Ma) by analyses using *rbc*L and fossil calibrations. Notably, in that study the family was represented by only 16 terminals and there was no internal calibration point. This could be improved by better sampling and by incorporating internal fossil calibration points [12]. Kato et al (2003) [13] dated the seagrasses within Hydrocharitaceae at 119 ± 11 Ma by analyses using the substitution rates of *rbc*L and *mat*K. However, this time overlaps with the generally accepted age of the order Alismatales thus putting the validity of the results of that study into doubt [14]. He et al. (1991) [15] proposed that *Ottelia *had originated no later than the Cretaceous based on the distribution of the genus and the predictions of the continental drift theory. Fossils of Hydrocharitaceae have been found in Europe including those of the extant genera *Vallisneria, Hydrilla, Ottelia, Thalassia, Stratiotes, Hydrocharis *and *Najas *from the Eocene, Oligocene and Miocene [16-18]. The oldest fossil of Hydrocharitacae (genus *Stratiotes*) is from the Late Paleocene [19]. These fossils have prior to the present study not been incorporated in divergence time estimates.

The geographic origin of Hydrocharitaceae remains unresolved. The diversity centre of the family has been suggested to be in tropical Asia [20]. However, the diversity centre of a taxon does not necessarily correspond to its centre of origin. Numerous fossils of Hydrocharitaceae have been found in Europe [17], implying a possible European origin of the family. A biogeographic analysis is required to elucidate the geographical origin of the family.

The transoceanic distribution of angiosperms has long intrigued biologists. Two competing hypotheses have been proposed to explain this phenomenon: the first attributing it to dispersal [21-23] and the second to vicariance (continental drift) [24]. Les et al. (2003) [21] proposed that dispersal is the major factor accounting for the disjunctive distribution of aquatic plants. This is contrary to the traditional viewpoint which considered vicariance [24] as the major cause of the disjunctive distribution in aquatic taxa such as Limnocharitaceae [25], *Ottelia *(Hydrocharitaceae) [15] and *Sagittaria *(Alismataceae) [26]. Hydrocharitaceae exhibits a wide transoceanic distribution at genera and species levels. The family can serve as a suitable model to investigate transoceanic distribution in aquatic monocots.

About 10% of all flowering plants have unisexual flowers which have traditionally been regarded as a derived state in angiosperms [27]. Most species of Hydrocharitaceae are unisexual while some are hermaphrodite. Hermaphroditism is regarded as the ancestral condition which gave rise to unisexual flowers [28]. However, hermaphroditic flowers also occur in more recently evolved genera such as *Ottelia *[1]. This suggests that the view that hermaphroditism is the ancestral state needs to be re-examined. Leaf habit in Hydrocharitaceae varies from aerial, aerial-submerged to fully submerged, and leaf shape varies from circular, linear, to ribbon like [1]. Ancestral state reconstruction is useful in understanding the evolutionary history of reproductive system and leaf morphology and their significance in adapting the plants to the aquatic environment.

In this study, we sampled 17 genera of Hydrocharitaceae, using DNA sequences from 8 genes. The aims of our study were: 1) to reappraise the generic relationships of Hydrocharitaceae; 2) to estimate the divergence times; 3) to investigate the area of origin and the major factor(s) shaping the global distribution; and 4) to investigate the evolution of reproductive system and leaf morphology in this family.

## Results

### Sequence characteristics

Details of the voucher and DNA sequence information are provided in Additional file 1. We successfully generated 117 sequences. Other sequences used in this study were downloaded from GenBank. Seventeen of the 18 genera in Hydrocharitaceae were sampled. Genus *Appertiella *was not included because of unavailability of molecular data. The supermatrix dataset, which resulted from assembling the DNA sequences of the 8 genes, was 8,086 nt in length (2,035 mt, 4,406 chl, 1,645 nu). The dataset consisted of 38 terminals of Hydrocharitaceae and was submitted to TreeBASE (accession number S12110). The dataset comprised about 29% missing characters mainly due to unavailability of some sequences.

### Phylogenetic analyses

**Figure 1:**
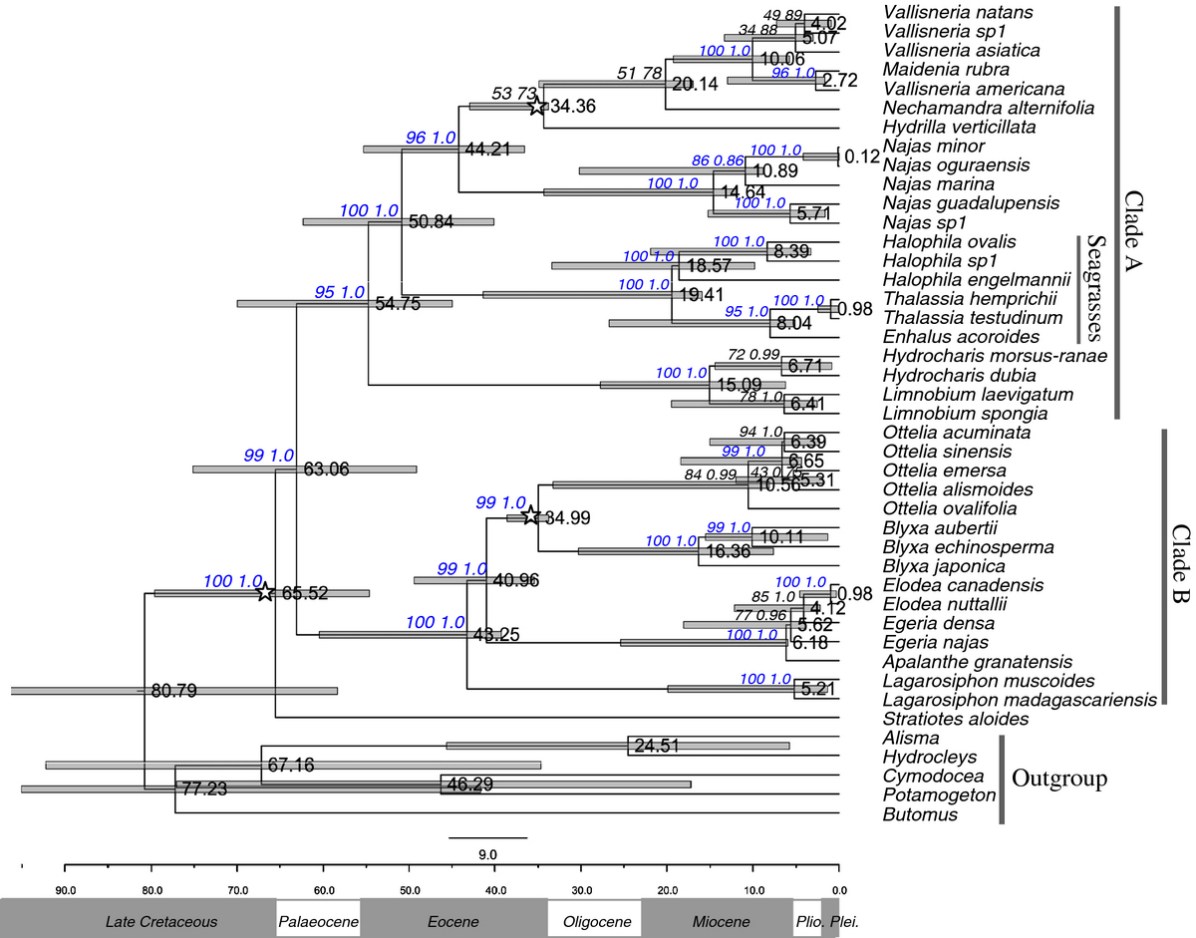
**Phylogeny and divergence time estimates of Hydrocharitaceae based on combined 18S + *rbc*L+ *mat*K+ *trn*K 5' intron + *rpo*B+ *rpo*C1 + *cob *+ *atp*1 data set**. Numbers above branches refer to the maximum likelihood bootstrap values (BS, left) and the posterior probabilities (PP, right). Numbers in blue refer to the branches with BS> 95 and PP = 1.0. Gray bars indicate 95% highest posterior distributions, and nodes labelled with stars refer to the positions of fossil calibration points.

*Butomus *and (*Butomus *+ *Alisma *(Alismataceae) + *Cymodocea *(Cymodoceaceae) + *Hydrocleys *(Limnocharitaceae) + *Potamogeton *(Potamogetonaceae)) were independently selected as outgroup. Both Maximum likelihood (ML) and Bayesian analysis using the supermatrix dataset resulted in completely identical relationships and strong support (bootstrap value (BS)> 95, Bayesian posterior probability (PP) = 1.0) for most branches (Figure 1). ML analysis involving partitioning the supermatrix dataset into eight genes and no partition resulted in slightly different support values, but identical topologies. *Stratiotes *was resolved as the first diverging lineage of Hydrocharitaceae with strong support (BS = 100, PP = 1.0). Other genera formed two clades (BS = 99, PP = 1.0; Figure 1). Clade A (BS = 95, PP = 1.0) included 10 genera. *Hydrocharis*-*Limnobium *was resolved as the first diverging lineage of this clade; the seagrasses formed a well supported clade (BS = 100, PP = 1.0) which was resolved as sister to the subclade (BS = 96, PP = 1.0) formed by *Najas, Hydrilla, Nechamandra, Vallisneria *and *Maidenia *(Figure 1). Clade B (BS = 100, PP = 1.0) consisted of *Lagarosiphon, Ottelia, Blyxa, Apalanthe, Elodea *and *Egeria *(Figure 1). The two outgroups selected resulted in slightly different support values, but identical topologies of Hydrocharitaceae.

### Dating analysis and ancestral area reconstruction

All BEAST MCMC runs yielded sufficient effective sample sizes (> 200) for all relevant parameters and converged on topologies identical to the tree in Figure 1. The crown node age of Hydrocharitaceae was dated at 65.2 Ma (95% HPD: 54.6-79.6 Ma). The mean divergence between clade A and clade B, estimated to be 63.1 Ma. For the three calibration nodes, mean posterior estimates were consistent with prior node ages, suggesting that the calibration points were sufficiently concordant [29,30].

**Figure 2:**
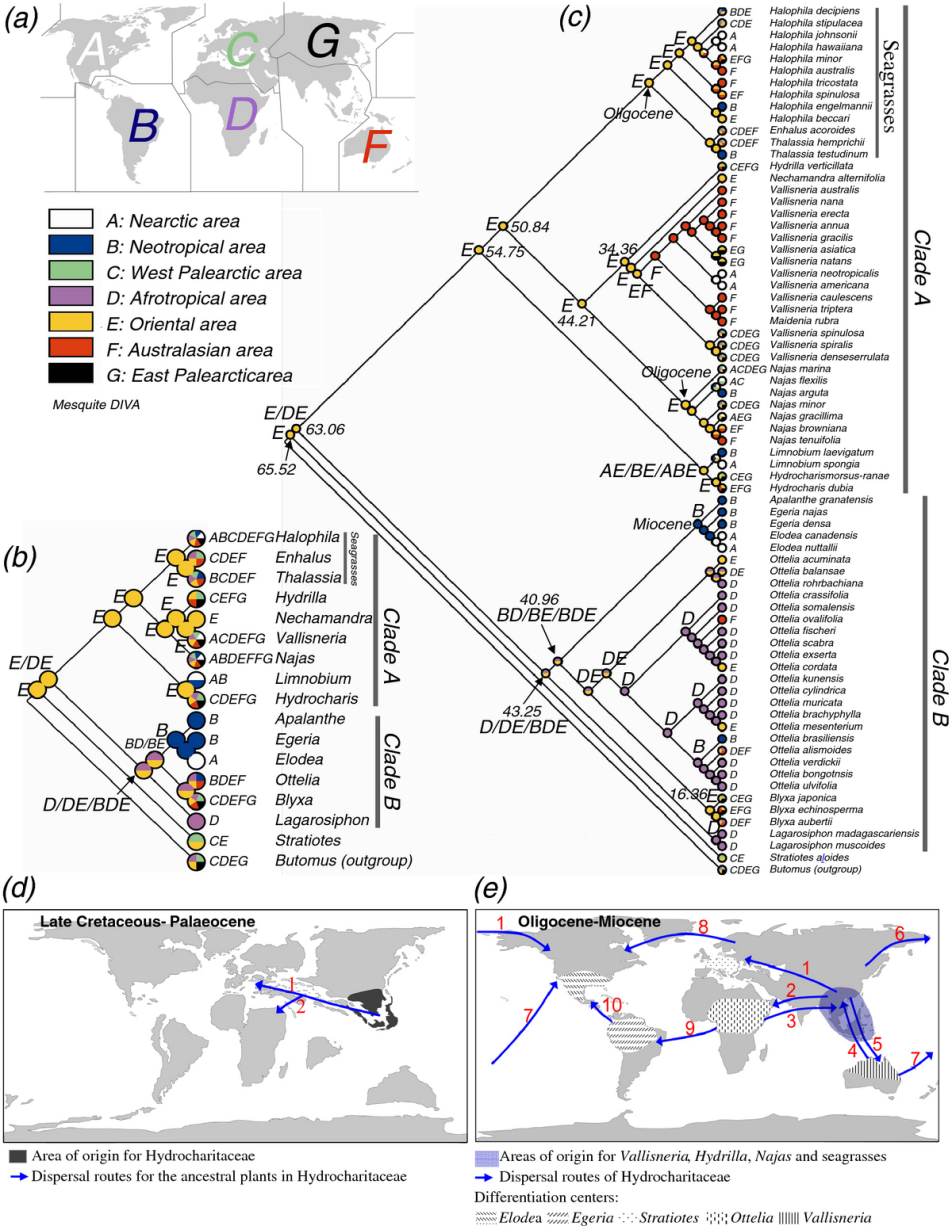
**Historical biogeography of Hydrocharitaceae**. (a) Area delimitation in biogeographic analyses. (b) Analyses using genera as terminal taxa, and (c) analyses using species as terminal taxa. Distribution of each genus or species is indicated along with taxon names. Results of Mesquite are indicated by coloured circles; results of dispersal-vicariance analysis (DIVA) are indicated by capital letters (only the results of major clades are shown); equally optimal ancestral distributions are indicated by pie-charts (Mesquite) or slashes (DIVA). The times inferred from divergence time estimates were marked on the major clades, numbers represent millions of years before present. (d) & (e) Possible origin, differentiation centres and dispersal routes of Hydrocharitaceae.

Seven biogeographic areas were recognized according to Morse [31] (Figure 2a) namely A, Nearctic area; B, Neotropical area; C, West Palearctic area; D, Afrotropical area; E, Oriental area; F, Australasian area; and G, East Palearctic area. Two strategies were applied in the biogeographic analyses. One used genera as terminal taxa, the other used species as terminal taxa. Results of the analyses using genera as terminal taxa suggested that the most recent common ancestors (MRCAs) of both Hydrocharitaceae and clade A occurred in Oriental area. The MRCA of clade B occurred in Oriental, Afrotropical and Neotropical areas (Figure 2b).

Results of the biogeographic analyses using species as terminal taxa suggested that Hydrocharitaceae originated in Orient (Figure 2c). A minimum of 76 dispersal events was inferred from DIVA to explain the current distribution of Hydrocharitaceae. The ancestor of *Stratiotes *was suggested to have been in Orient and dispersed to Europe during the Late Cretaceous and Paleocene (Figure 2c, d, arrow 1). This route is similar to that which has been reported for *Alangiopollis *(Alangiaceae) [32]. The analyses using genera or species as terminal taxa yielded comparable results on the ancestral area of Hydrocharitaceae. This indicates that incomplete sampling may have little effect on the accuracy of investigation into the geographical origin of Hydrocharitaceae. However, the analyses using species as terminal successfully resolved the ancestral areas for more genera than those using genera as terminal.

The MRCAs of clade A, the seagrass genera and the seagrass subclade were shown to be of Oriental origin. The taxa then diversified in Oriental region (DIVA, Mesquite; Figure 2c). DIVA suggested that *Vallisneria *originated in Oriental and Australasian regions. Mesquite suggested an Oriental origin for *Vallisneria*, followed by diversification and dispersal to other continents (Figure 2c). Long distance dispersal (LDD) for *Vallisneria *from Oriental area to Africa and Europe was inferred from *V*. *spinulosa, V*. *spiralis *and *V*. *denseserrulata *(Figure 2c, e, arrow 1 & 2). LDD from Australasia to North America for this genus was inferred from *V. neotropicalis *and *V. americana *(Figure 2c, e, arrow 7). This route is similar to that envisaged for the taxon *Scaevola *(Goodeniaceae) [33]. LDD from Australasia to Asia similar to that which has been recorded for the plant family Cucurbitaceae [34] was inferred from taxa including *V. asiatica *and *V. natans *(Figure 2c, e, arrow 4). The genus *Najas *was inferred to have originated in Oriental area during the Oligocene. LDD from Asia to North America (inferred from *N*. *gracillima*), to Europe (inferred from *N*. *minor*), to Africa (inferred from *N*. *minor*) and to Australia (inferred from subclade *N*. *browniana *+ *N*. *tenuifolia*) was suggested (Figure 2c, e, arrow 6, 1, 2 & 5).

The ancestor of clade B was inferred to have dispersed from Orient to Afrotropical region (Mesquite) or Southern hemisphere (DIVA) during the Eocene (Figure 2c, d, arrow 2), followed by diversification in Southern hemisphere during the Tertiary. MRCA of *Ottelia *was suggested to have lived in Oriental and Afrotropical regions (Figure 2c). LDD from West Africa into South America for this genus was inferred from *O*. *brasiliensis *(Figure 2c, e, arrow 9). This route is similar to the one suggested for the dispersal of *Gossypium *(Malvaceae) [35] and is further supported by the fact that *Ottelia *in South America is confined to the southeastern area [36]. LDD from S.E. Asia to Australasia was inferred from *O*. *ovalifolia *(Figure 2c, e, arrow 5). Dispersal from Africa into Asia was also inferred from *O*. *cordata *and *O*. *mesenterium *(Figure 2c, e, arrow 3). This route is similar to that envisaged for the two genera *Coccinia *and *Momordica *in family Cucurbitaceae [34]. The MRCA of the subclade comprising *Apalanthe, Egeria *and *Elodea *was suggested to have arisen in Neotropical region, while the ancestor of this subclade may have come from Afrotropical region (Figure 2c, e, arrow 9). Dispersal from South America to North America during the Miocene was inferred for the ancestor of *Elodea*, probably via the island chains of Central America (Figure 2c, e, arrow 10).

### Ancestral character state reconstructions

**Figure 3:**
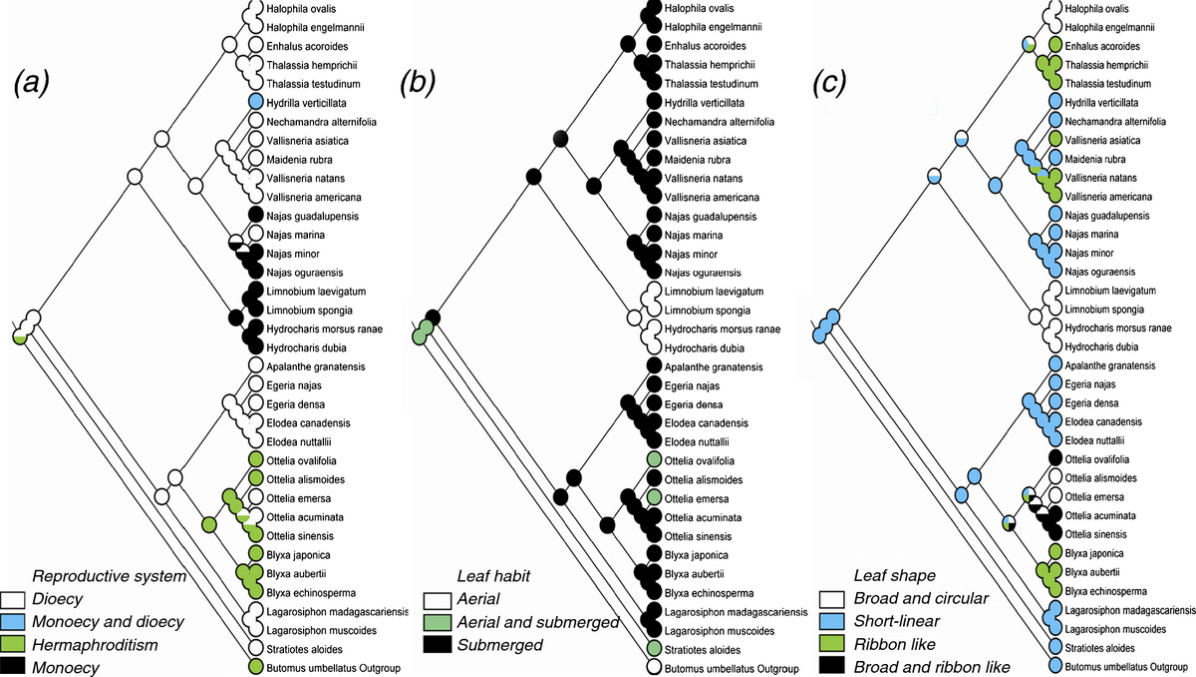
**Reconstruction of ancestral character states by Mesquite**.

Morphological characters of each species used in analysis were indicated in Figure 3. Dioecy was suggested as the progenitorial state, monoecy and hermaphroditism were derived from it (Figure 3a); *Ottelia emersa *and *O*. *acuminata *experienced reverse evolution from hermaphroditism to dioecy (Figure 3a). The aerial-submerged leaf habit was suggested as the progenitorial state, which gave rise to aerial leaf and submerged leaf (Figure 3b); the aerial-submerged leaf in *O*. *ovalifolia *and *O*. *emersa *was originated from the submerged leaf due to reverse evolution (Figure 3b). The short-linear leaf shape was suggested as the ancestral state, ribbon like and broad-ribbon like leafs were derived from it (Figure 3c); the broad-circular leaf evolved independently in three lineages, viz. *Hydrocharis*-*Limnobium, Ottelia *and *Halophila*.

## Discussion

### Systematics

By merging diverse sequences into a supermatix data set, we obtained a well-resolved phylogeny with most branches strongly supported by BT values greater than 95% and PP values of 1.0. This indicates that increasing the number of taxa and the number of molecular markers improved the resultant phylogeny, and it further supports the notion that a supermatrix can be used to obtain a well-resolved and strongly supported phylogeny in cases where some data are missing [37-39]. The phylogenetic relationships of *Egeria, Elodea, Ottelia, Blyxa, Apalanthe *and *Lagarosiphon *have remained unchanged in all the earlier molecular phylogenetic studies [1,2,8,9,40,41]. Our analyses resolved the generic relationships that are largely similar to those reported in those studies. However, incongruences existed for the other genera, which we briefly address here below.

The genus *Hydrilla *comprises only one species, *H. verticillata*. Based on *rbc*L, *mat*K, *trn*K intron, ITS and morphological data, Les et al. (2006) [1] suggested that *Hydrilla *was most closely related to *Najas*, despite their being quite divergent at the phenotypic level. Our phylogenetic analyses suggested that *Hydrilla *is most closely related to the subclade comprising *Nechamandra, Vallisneria *and *Maidenia *(Figure 1). This position is consistent with all previous phylogenetic studies (except Les et al., 2006) based on molecular and morphological data [2,8,9,40,41]. A close relationship between the subclade (*Hydrilla *(*Nechamandra *(*Vallisneria *+ *Maidenia*)) and *Najas *was strongly supported (BS = 96, PP = 1.0, Figure 1). This is in agreement with earlier results from *rbc*L+ *mat*K+ *trn*K intron analyses with ML approach [1], *rbc*L [9], and *rbc*L+ *mat*K [8]. However, the results did not support the close affinity between the subclade and seagrasses which had been inferred from *rbc*L [2] and *rbc*L+ *mat*K+ *trn*K intron + ITS analysis [1].

*Stratiotes *was resolved as the first diverging lineage within Hydrocharitaceae (BS = 100, PP = 1.0; Figure 1). This is in agreement with analyses based on *rbc*L [9], *mtt*2 and *nad*5 [10] and the fossil records of *Stratiotes *which include the most abundant and the oldest fossils of' Hydrocharitaceae [19,42]. However, the phylogenetic position of *Stratiotes *seems to be mainly derived from the mitochondrial sequences (*cob, atp*1) which are prone to flaws in plant phylogenetic analysis [43]. Therefore, further studies are required to confirm the position of the genus obtained in this study.

### Origin

Results of divergence time estimates are in agreement with the fossil records of Hydrocharitaceae. The 95% HPD of *Najas *was 11.9-34.3 Ma, consistent with the oldest fossil of this genus in the Oligocene [17] (Figure 1). The stem node age of *Hydrocharis*-*Limnobium *was dated around 54.7 Ma. However, the crown node age of this subclade was dated around 15.9 Ma, younger than the oldest fossil of *Hydrocharis *from the Upper Eocene [17,44,45]. This could be interpreted as an indication that *Limnobium *had split from the relatively ancient *Hydrocharis *in the Miocene (Figure 1), and the great morphological similarity between the two genera is probably due to the short evolutionary history of *Limnobium*. Although the present study has yielded improved divergence time estimates, it is possible that the estimates of the time of origin for some genera such as *Ottelia, Vallisneria, Najas *and *Blyxa *may have been affected by under-representation in sampling.

The age of Hydrocharitaceae estimated in this study (mean: 65.5 Ma, 95% HPD: 54.6-79.6 Ma) is in agreement with that based on *rbc*L analysis and external fossil calibration points (crown node age = 75 Ma) [11]. However, the stem node age of seagrasses estimated in this study (15.9-41.3 Ma) (Figure 1), is more recent than the 119 ± 11 Ma suggested from analysis using the substitution rates of *rbc*L and *mat*K [13]. Similarly, our estimates of the stem node age of *Ottelia *(8.1-33.3 Ma) is more recent than the Cretaceous origin suggested by He et al. (1991) [15]. The split between Zosteraceae and Potamogetonaceae has been dated at 47 Ma by *rbc*L and fossil calibration [11]. The time is also more recent than the 100 Ma inferred from analyses using the substitution rates of *rbc*L and *mat*K [13]. These discrepancies indicate that for estimating divergence times in aquatic plants, incorporating fossil calibration point would be more reliable.

The Oriental origin of Hydrocharitaceae inferred from our analysis is supported by the known existence of regions with humid and warm conditions in southeastern Asia during the late Cretaceous and Palaeocene [46,47] and the fact that the genetic diversity centre of this family is in tropical Asia [20]. The ancestor of clade A was inferred to have originated and diversified in the Orient, while that of clade B dispersed from the Orient to the Southern Hemisphere during the Late Cretaceous and Paleocene (Figure 2c, d, arrow 2). Different environments and oceanic barriers among the major continents (vicariance mechanism) during the Tertiary probably contributed to the diversification of this family resulting in taxa such as the African endemic *Lagarosiphon*.

Most fossils of Hydrocharitaceae and its close relatives Butomaceae and Alismataceae have been found in Europe (Butomaceae in the Neogene of south Aral region, Miocene of northwest and east Caucasus [18,48]; Alismataceae in the Tertiary of Europe, a few in North America). The fossil records seem to be inconsistent with the Oriental origin of this family. However, the absence of reports of fossils from Asia most likely reflects a bias in paleobotany, rather than an indication of the origin and past distribution of Hydrocharitaceae. A similar situation exists in *Rhinolophus *(Rhinolophidae), for which, although the genus is thought to have originated in Asia, fossils have only been reported from Europe and Africa but not from Asia [49,50].

### Does the origin fit with dispersal?

The modern continents viz. South America, Africa, Eurasia, Australia and North America have been separated by oceans since at least ca. 90 Ma [46,51,52], earlier than the origin of Hydrocharitaceae. Therefore, dispersal must have played a dominant role in the transoceanic distribution of this family. This contradicts the view that the transoceanic distribution of *Ottelia *mainly resulted from vicariance [15]. The role of dispersal in transoceanic distribution has been supported by evidences from the studies of geological events and land plant families. Ocean currents are a viable means of dispersal of plants [53], and a tropical westward-flowing ocean current had spanned the world from the Cretaceous to Paleocene [54,55]. Island chains existed in the Tethys from Cretaceous to Eocene, which served as a stepping-stone in biotic dispersal between S.E. Asia, Africa and southern Europe [32,56,57]. The Malay Archipelago probably facilitated biotic dispersal between S.E. Asia and Australia during the Miocene [58]. The North Atlantic Land Bridge (NALB) aided plant migration between North America and Europe during the late Cretaceous and early Tertiary [59-61]. The Bering Land Bridge (BLB) was open from at least the early Paleocene until its closure ca. 7.4-4.8 Ma [62]. Several recent studies of angiosperms based on molecular and fossil data have supported dispersal as the dominant factor responsible for transoceanic distribution, e.g., in Cucurbitaceae [34], Sapindaceae [63], Chrysophylloideae (Sapotaceae) [64], Burseraceae [65] and Malphigiaceae [52]. It is probable that Hydrocharitaceae have dispersed to all continents of the world via island chains, land bridges and ocean currents.

Biogeographic studies have suggested that the sub-cosmopolitan distribution of the aquatic plant family Alismataceae has mainly resulted from dispersals (the work will be reported in a separate paper). It is probable that dispersal is the dominant factor, accounting for transoceanic distribution of aquatic angiosperms. However, more studies on aquatic angiosperms are required to investigate this idea further.

### Historical biogeography of some genera of Hydrocharitaceae

The ancestor of *Stratiotes *was suggested to have dispersed from Orient into Europe during the late Cretaceous and Palaeocene (Figure 2b &2c, 2d, arrow 1), which coincided with the existence of the Tethys seaway (TESW) [54]. Alternatively, the ancestor may have migrated from Orient to Europe across Eurasia. Abundant fossils (15 fossil species) of this genus in Europe [42] suggested that the genus had diversified widely in this region adapting to wet swamps in the Late Cretaceous [66].

The genus *Hydrilla *is native to Eurasia and Australia [67], and introduced to Americas [68] and parts of Africa [69]. The centre of differentiation of the genus was thought by Cook and Luond (1982) to lie in tropical Asia [70]. This idea got support from genetic diversity analysis which revealed that the highest diversity is located in China and with lower albeit similar genetic types occurring in Africa, India and USA [71]. *Hydrilla *might have arisen in the Orient dispersing to Europe and Australia (Figure 2, e, arrow 1 & 5).

The MRCA of the seagrasses within Hydrocharitaceae were suggested to have lived in Oriental area during the Oligocene and Miocene. The result is in agreement with the view that seagrasses possibly originated in the S.E. Asia [72,73]. The result is supported by the environments of S.E. Asia which was characterized by abundant islands, spacious shallow-seas, warm temperature and plenty of isolated seas [74]. However, the result denied the Cretaceous origin of the group which has been suggested in previous studies [13,75,76]. The seagrasses were suggested to have been dispersed from Oriental to other regions (Figure 2c), probably by ocean currents [73,77]. For example, the warm northward Kuroshio Current carried seagrasses from the equatorial region to the Nansei Islands [77]. Seagrasses are capable of surviving during the LDD between major ocean systems [78].

*Vallisneria *has a world-wide distribution, with the highest number of species in Australia [79,80]. Les et al. (2008) [80] resolved the phylogeny of this genus, but they conceded that the geographical origin is difficult to pinpoint. In this study by DIVA analysis, Oriental and Australasian areas were suggested as the co-existed ancestral areas of *Vallisneria*. However, Oriental area is more likely the centre of origin considering the following facts: the closest relative of *Vallisneria *namely *Nechamandra *is confined to Asia [67]; the ancestral species in *Vallisneria *namely *Vallisneria spinulosa, V*. *spiralis *and *V*. *denseserrulata *are confined to the Old World [67,80].

### Evolution of morphological characters

Ancestral state reconstruction of reproductive system in Hydrocharitaceae provides empirical evidence that evolution of dioecy in plants has been a bidirectional, viz. from dioecy to hermaphroditism, and from hermaphroditism to dioecy (Figure 3a). This view is supported by Delph (2009) [81] and Canovas et al. (2011) [82], but rejects the view that hermaphroditism is the ancestral state in Hydrocharitaceae [28].

The evolution of leaf habit and leaf shape in Hydrocharitaceae provides several cases of evolutionary adaptation to diverse habitats. The evolution from aerial-submerged leaf to submerged leaf is probably due to change in habitat from shallow to deep waters [83]. The reverse evolution from submerged leaf to aerial-submerged leaf in *Ottelia *is probably an adaptation to change in habitat from deep to shallow water or some other disadvantageous habitat(s). Taxa with broad-circular leaves (e.g., *Ottelia *and *Hydrocharis*) usually occur in still water, while those with ribbon like leaves such as *Enhalus *and *Thalassia *occur in coastal waters with strong waves [67].

## Conclusions

In summary, this study has reconstructed the phylogeny of Hydrocharitaceae. The family was suggested to originate in Oriental area during the Late Cretaceous and Paleocene (54.7-72.6 Ma). Dispersal is the most likely factor shaping the transoceanic distribution of this family. Ancestral character state reconstruction of gender and leaf morphology offered valuable information for understanding adaptive evolution in aquatic monocots. However, the historical biogeography for some genera (e.g., *Ottelia, Vallisneria*) may suffer from under-representation in sampling, and require re-evaluation in future studies.

## Methods

### Sampling and molecular protocols

Most materials used for DNA sequencing was collected from Wuhan Botanical Garden. Some were collected from natural populations in China. Eight genes were used, among which 18S is from nuclear; *rbc*L, *mat*K, *trn*K5' intron, *rpo*B and *rpo*C1 are from chloroplasts; *cob *and *atp*1 are from mitochondria. A detailed list including the voucher information and GenBank accession numbers is provided in Additional file 1.

Genomic DNA was extracted from silica-dried leaves using the Plant Genomic DNA Isolation Kit (Dingguo Biotech, Beijing, China). All polymerase chain reactions (PCR) were conducted in the ABI 2720 Thermal Cycler (Applied Biosystems) in 40 μl volume containing 4 μl of 10 × amplification buffer (200 mM Tris-HCl (pH 8.4), 200 mM KCl, 100 mM (NH_4_)_2_SO_4_, 20 mM MgSO_4_), 0.8 μl of each primer (10 μM), 0.8 μl of dNTPs (10 mM), 2 U of Taq DNA polymerase (TransGen Biotech Co., Beijing, China) and 60 ng of DNA template. For *cob, atp*1, 18S, *trn*K 5' intron, *rpo*B and *rpo*C1, the following PCR profile was adopted: 94°C for 3 min, 35 cycles of 30 s at 94°C, 30 s at 50°C, 1 min at 72°C and a final step for 10 min at 70°C. For *mat*K, 55°C Tm and 1 min 30 s extension times were used. Primer sequences were obtained from previous studies: *cob *(COB1F, COB1R) and *atp*1 (atpAF1.5, atpAl137r) [9]; *rbc*L (1 F, 1204R) [84]; *mat*K [7]; *trn*K 5' intron (3914-F, TRANK2-R) [85]; 18S (N-NS1, C-18 L) [86]; *rpo*B (1f, 4r) and rpoC1 (2f, 4r) [87]. Purified PCR products were double direction sequenced using an automated DNA sequencer (ABI 3730, Applied Biosystems). All newly generated sequences were deposited in GenBank (Additional file 1).

### Phylogenetic analyses

All sequences were aligned individually using Clustal X v2.0 [88]. The output was manually inspected, and ambiguously aligned parts were excluded. Inspired by other studies (e.g. [37,39,89,90]), we assembled all the aligned sequences into a supermatrix data set (combined data set), which was used in phylogenetic analyses. *Butomus *(Butomaceae) was used as outgroup according to Les et al. (2006) [1]. We also selected (*Butomus *+ *Alisma *+ *Cymodocea *+ *Hydrocleys *+ *Potamogeton*) as outgroup in order to investigate the influence of outgroup in topology of Hydrocharitaceae.

ML analysis was conducted using RAxML v7.2.5 [91] via the Cyberinfrastructure for Phylogenetic Research (CIPRES) Portal http://www.phylo.org. Two strategies were employed, one involved partitioning the supermatrix data set into eight genes, while another did not partition the data set. GTRCAT and GTRGAMMA options were used, 1000 rapid bootstrap replicates were conducted to assess bootstrap values.

Bayesian analysis was conducted in MrBayes v3.1.2 [92]. The best-fit model of nucleotide substitution was chosen by MrModeltest v2.3 [93] according to the Akaike Information Criterion (combined data set: GTR + I + G model). Two separate runs of four concurrent runs (one cold, three heated each) of 16,000,000 generations were employed with sampling at every 1,000 generations. The stationarity of the likelihood scores of sampled trees was evaluated in Tracer v1.5 [94], and the first 10% generations were discarded as burn-in.

### Divergence time estimates

Divergence time estimates were conducted in BEAST v1.5.4 [95] using the supermatrix data set. *Butomus, Alisma, Cymodocea, Hydrocleys *and *Potamogeton *were selected as outgroup. To prevent the negative effects from heterogeneity of substitution rates and uncertainty of fossil data, we used a relaxed clock and Uncorrelated Lognormal (UCLN) model [96,97]. GTR + I + G model with Gamma Categories set to 6 was adopted. The starting tree was randomly generated with a Yule process prior. More than 90,000,000 generations of MCMC were implemented of which every 1,000 generations were sampled. Tracer v1.5 [94] was used to check the parameters and the first 10% generations were discarded as burn-in.

Lognormal distribution was selected for each calibration point according to Adamson et al. (2010) [98]. This distribution defined the minimum ages for calibrated nodes but allowed the maximum ages to be estimated following a lognormal distribution without hard limit [99]. Three calibration points were incorporated. The oldest reliable fossils of *Hydrilla *and *Vallisneria *were reported from the Upper Eocene (33.7-55.8 Ma) [17,44]. Therefore, the split between *Hydilla *and (*Vallisneria *+ *Nechamandra*) was constrained to a minimum of 33.7 Ma (offset = 33.7, mean = 1.1, SD = 1.2). In addition, the oldest reliable fossil of *Ottelia *was from the Upper Eocene (33.7-55.8 Ma) [17,44]. Therefore, the split between *Blyxa *and *Ottelia *was constrained to a minimum of 33.7 Ma (offset = 33.7, mean = 1.1, SD = 1.2). Lastly, the oldest fossil of this family (genus *Stratiotes*) was 0.1 Ma younger than the Paleocene-Eocene boundary (54.6 Ma) [19]). Therefore, the fossil was used to set the split between *Stratiotes *and the remaining genera of this family not later than 54.5 Ma (offset = 54.5, mean = 1.0, SD = 1.0).

### Biogeographic analyses

Seven biogeographic areas were recognized according to Dr Morse [31] (Figure 2a). Biogeographic distribution of Hydrocharitaceae was mainly compiled from literature [36,67,72,78,80,100-106]. Fossil was not considered in the area coding as no fossil has been found outside the natural distribution of any genus. Distribution that is known to have been caused by human activities was not included in the analyses. Two methods were used in the analyses: dispersal-vicariance analysis implemented in DIVA v1.2 [107] with the maximum number of ancestral areas at each node constrained to four, and parsimony ancestral state reconstruction implemented in Mesquite [108].

Two strategies were applied in the biogeographic analyses. One strategy used genera as terminal taxa in the analyses. A tree (Figure 2b) that represented the generic topologies inferred from the phylogenetic analysis using the supermatrix (Figure 1) was constructed, and it was used in the analyses. Each genus was coded based on the current distribution. Details of the distribution are provided in Figure 2b. This strategy followed the methods in the biogeography study of Ranunculaceae [23] and the suggestion from Yan Yu (one of the authors of S-DIVA). Another strategy used species as terminal taxa and a tree including 72 species of Hydrocharitaceae was used (Figure 2c). Species belonging to *Ottelia *[15], *Halophila *[78] and *Vallisneria *[80] with known phylogenetic relationships were manually added to the tree which resulted from the phylogenetic analysis (Figure 1). In addition, seven species of *Najas *with the topology from a ML analysis based on *rbc*L were also added to the tree. Each species was coded based on the current distribution. Details of the distribution are provided in Figure 2c. The purpose of using species as terminal taxa was to reconstruct the ancestral areas at the family and genus levels.

### Ancestral character state reconstructions

Information on the reproductive system, leaf habit and leaf shape was mainly compiled from literature [1,36,67,103]. Details of the phenotypic data are provided in Figure 3. Parsimony ancestral state reconstruction was performed using the Mesquite [108] and the tree inferred from the phylogenetic analyses using the supermatrix dataset.

## Competing interests

The authors declare that they have no competing interests.

## Authors' contributions

LYC participated in design of the study, carried out the experiment work, performed data analyses and drafted the manuscript. JMC participated in design of the study and helped to draft the manuscript. GWR revised the manuscript. QFW conceived the study, revised the manuscript and gave final approval of the version to be published. All authors read and approved the final manuscript.

## Appendix A

Additional file 1 Taxa included in this study with voucher information and GenBank accession numbers (DOC 99 kb)

## Supplementary Material

Additional file 1 **Taxa included in this study with voucher information and GenBank accession numbers (DOC 99 kb)**. Click here for file
