# Supplementary material for: Generic phylogeny, historical biogeography and character evolution of the cosmopolitan aquatic plant family Hydrocharitaceae
Source: BMC Evol Biol. 2012 Mar 10;12:30. doi: 10.1186/1471-2148-12-30 (PMC3317846; doi:10.1186/1471-2148-12-30)
Supplement: Additional file 1 — Taxa included in this study with voucher information and GenBank accession numbers (DOC 99 kb). [file 1471-2148-12-30-S1.DOC]

### Additional file 1–Taxa included in the present study with voucher information and GenBank accession numbers.

*, sequences generated in current study. -, missing sequences. Most material which collected from Wuhan Botanical Garden was originally from the natural populations of China. *Elodea Canadensis* and *Egeria densa* were originally collected from invasive populations in China. *Stratiotes aloides* was originally bought from a store in Liaoning province, China. *Hydrocleys nymphoides* was originally collected from the United States.

| Taxon | Voucher information | 18S | *rbc*L | *mat*K | *trn*K 5’ intron | *rpo*B | *rpo*C1 | *cob* | *atp*1 |
| --- | --- | --- | --- | --- | --- | --- | --- | --- | --- |
| *Vallisneria asiatica* | Botanical Garden, Wuhan, China. 06, 2010 | AY952384 | EF155532 | AY957575 | JF975503* | JF975529* | JF975547* | JF975464* | JF975481* |
| *Vallisneria natans* | Botanical Garden, Wuhan, China. 06, 2010 | JF975494* | JF975497* | - | JF975504* | JF975530* | JF975548* | JF975465* | JF975482* |
| *Vallisneria americana* | GenBank | AF069201 | U03726 | AY870366 | AY870380 | - | - | - | - |
| *Vallisneria sp1* | GenBank | AF207050 | DQ859177 | - | - | - | - | DQ859158 | DQ859119 |
| *Egeria densa* (*Elodea densa*) | Botanical Garden, Wuhan, China. 06, 2010 | JF975484* | AB004887 | AB002567 | AY870385 | JF975514* | JF975532* | - | JF975467* |
| *Egeria najas* | GenBank | - | DQ859166 | - | - | - | - | DQ859134 | DQ859098 |
| *Limnobium laevigatum* | GenBank | - | AB004894 | AB002574 | - | - | - | DQ859141 | DQ859105 |
| *Limnobium spongia* | GenBank | - | U80704 | - | AY870376 | - | - | - | - |
| *Hydrilla verticillata* | Botanical Garden, Wuhan, China. 06, 2010 | JF975488* | U80700 | AB002571 | AY870378 | JF975518* | JF975536* | JF975454* | JF975474* |
| *Elodea canadensis* | Botanical Garden, Wuhan, China. 06, 2010 | AF168841 | DQ859167 | - | JF975505* | JF975515* | JF975533* | DQ859135 | DQ859099 |
| *Elodea nuttallii* | GenBank | - | AB004888 | AB002568 | AY870386 | - | - | - | DQ508946 |
| *Ottelia alismoides* | Botanical Garden, Wuhan, China. 06, 2010 | JF975490* | AB004895 | AB002575 | AY870383 | JF975520* | JF975541* | JF975459* | JF975476* |
| *Ottelia acuminata* | Botanical Garden, Wuhan, China. 06, 2010 | AY952392 | AY952435 | AY952432 | JF975507* | JF975521* | JF975542* | JF975460* | JF975477* |
| *Ottelia ovalifolia* | GenBank | - | DQ859171 | - | - | - | - | DQ859146 | AY277802 |
| *Ottelia emersa* | Guiguang, Guangxi, China. 09, 2010 | JF975491* | JF975498* | JF975500* | JF975511* | JF975522* | JF975543* | JF975461* | JF975478* |
| *Ottelia sinensis* | Guiguang, Guangxi, China. 09, 2010 | JF975492* | JF975496* | JF975501* | JF975508* | JF975523* | JF975544* | JF975462* | JF975479* |
| *Blyxa echinosperma* | Botanical Garden, Wuhan, China. 06, 2010 | JF975483* | AB088810 | AB088781 | JF975509 | JF975513* | JF975531* | JF975451* | JF975466* |
| *Blyxa aubertii* | GenBank |  | U80694 | - | AY870384 |  |  | DQ859128 | DQ859093 |
| *Blyxa japonica* | GenBank | AY952406 | AB004886 | AB002566 | - | - | - | - | - |
| *Nechamandra alternifolia* | GenBank | - | U80706 | - | - | - | - | DQ859145 | DQ859109 |
| *Apalanthe granatensis* | GenBank | - | U80693 | AY870367 | AY870387 | - | - | - | - |
| *Thalassia hemprichii* | Lingshui, Hainan, China. 08, 2010 | AY952386 | U80710 | AB002577 | JF975510* | JF975528* | JF975546* | JF975463* | JF975480* |
| *Thalassia testudinum* | GenBank | AF168878 | U80711 | - | AY870373 | - | - | DQ859155 | DQ859117 |
| *Stratiotes aloides* | Liaoning, China. 04, 2011 | JF975493* | U80709 | AB002576 | AY870382 | JF975527* | JF975545* | DQ859153 | DQ859115 |
| *Lagarosiphon madagascariensis* | GenBank | - | AB004893 | AB002573 | - | - | - | - | - |
| *Lagarosiphon muscoides* | GenBank | - | U80702 | AY870368 | AY870388 | - | - | - | - |
| *Enhalus acoroides* | Lingshui, Hainan, China. 08, 2010 | AY952403 | U80697 | AB002569 | AY870372 | JF975516* | JF975534* | JF975452* | JF975469* |
| *Halophila ovalis* | Lingshui, Hainan, China. 08, 2010 | AY952400 | AB004890 | AB002570 | JN003598* | JF975517* | JF975535* | JF975453* | JF975473* |
| *Halophila sp1* | GenBank | - | DQ859168 | - | - | - | - | DQ859136 | DQ859100 |
| *Halophila engelmannii* | GenBank | - | U80699 | - | AY870374 | - | - | - | - |
| *Hydrocharis dubia* | Xingkaihu, Heilongjiang, China. 07, 2009 | JF975489* | JN578090* | AB002572 | JN003599* | JF975519* | JF975537* | JF975455* | JF975475* |
| *Hydrocharis morsus-ranae* | GenBank | - | U80701 | - | AY870375 | - | - | DQ859138 | DQ859102 |
| *Maidenia rubra* | GenBank | - | AY870370 | - | AY870379 |  | - | - | - |
| *Najas marina* | Botanical Garden, Wuhan, China. 06, 2010 | JF975485* | U80705 | JN003597* | AY870377 | JF975525* | JF975539* | JF975456* | JF975470* |
| *Najas oguraensis* | Botanical Garden, Wuhan, China. 06, 2010 | JF975486* | JF975499* | JF975502* | JF975506* | JF975526* | JF975540* | JF975457* | JF975471* |
| *Najas guadalupensis* | GenBank | AH001711 | DQ859169 | - | - | - | - | DQ859143 | DQ859107 |
| *Najas sp1* | GenBank | - | - | - | - | - | - | DQ859144 | DQ859108 |
| *Najas minor* | Botanical Garden, Wuhan, China. 06, 2010 | JF975487* | AB004899 | AB002579 | JF975512* | JF975524* | JF975538* | JF975458* | JF975472* |
| *Butomus umbellatus* | Changchun, Jilin, China. 07, 2009 | AH003489 | AY149345 | DQ401367 | JF781027* | JF781091* | JF781106* | DQ916649 | HQ31797 |
| *Alisma plantaga*-*aquatica* | Jiansanjiang, Heilongjiang, China. 07, 2009 | JF975495* | L08759 | AF542573 | JF781025* | JF781089* | JF781104* | DQ859125 | AF197717 |
| *Cymodocea rotundata* | Lingshui, Hainan, China. 08, 2010 | JN034102* | JQ031763* | JQ031760* | JQ031764* | JF781091* | JF781106* | JQ031761* | JQ031762* |
| *Hydrocleys nymphoides* | Botanical Garden, Wuhan, China. 06, 2010 | AY952397 | U80716 | AB002580 |  | JF781096* | JF781111* | DQ859139 | DQ859103 |
| *Potamogeton* | Genbank | AY952389 | DQ859173 | AB088780 | GQ247501 | AB559936 | JN034090* | DQ859150 | DQ859113 |
